# Supplementary material for: Polyphosphonate covalent organic frameworks
Source: Nat Commun. 2024 Sep 9;15:7862. doi: 10.1038/s41467-024-51950-1 (PMC11385950; doi:10.1038/s41467-024-51950-1)
Supplement: Supplementary file 1 — Supplementary Information [file 41467_2024_51950_MOESM1_ESM.pdf]

Supplementary Information

Polyphosphonate Covalent Organic Frameworks

Ke Xu,[‡1] Robert Oestreich,[‡2] Takin Haj Hassani Sohi,[‡2] Mailis Lounasvuori,[3] Jean G. A.Ruthes,[4,5] Yunus Zorlu,[6] Julia Michalski,[2] Philipp Seiffert,[2] Till Strothmann,[2] Patrik Tholen,[7] A. Ozgur Yazaydin,[8] Markus Suta,[2] Volker Presser,[4,5] Tristan Petit,[3] Christoph Janiak,[2] Jens Beckmann,[9] Jörn Schmedt auf der Günne[1], Gündoğ Yücesan[2]**

1. Department of Chemistry and Biology, Inorganic Materials Chemistry, University of Siegen, Adolf-Reichwein-Straße 2, 57076 Siegen, Germany
2. Institut für Anorganische Chemie und Strukturchemie, Heinrich-Heine-Universität Düsseldorf, Universitätsstraße 1, 40225 Düsseldorf, Germany
3. Young Investigator Group Nanoscale Solid-Liquid Interfaces, Helmholtz-Zentrum Berlin für Materialien und Energie GmbH, Albert-Einstein-Straße 15,12489 Berlin, Germany
4. INM – Leibniz Institute for New Materials, Campus D22, 66123 Saarbrücken, Germany.
5. Department of Materials Science and Engineering, Saarland University, Campus D22, 66123 Saarbrücken, Germany.
6. Department of Chemistry, Gebze Technical University, Kocaeli, Türkiye.
7. Technische Universität Berlin, Lebensmittelchemie und Toxikologie, Gustav-Meyer-Allee 25, 13355 Berlin, Germany
8. Department of Chemical Engineering, University College London, London WC1E 7JE, United Kingdom
9. Institut für Anorganische Chemie und Kristallographie, Universität Bremen, Leobener Str. 7, Bremen 28359, Germany

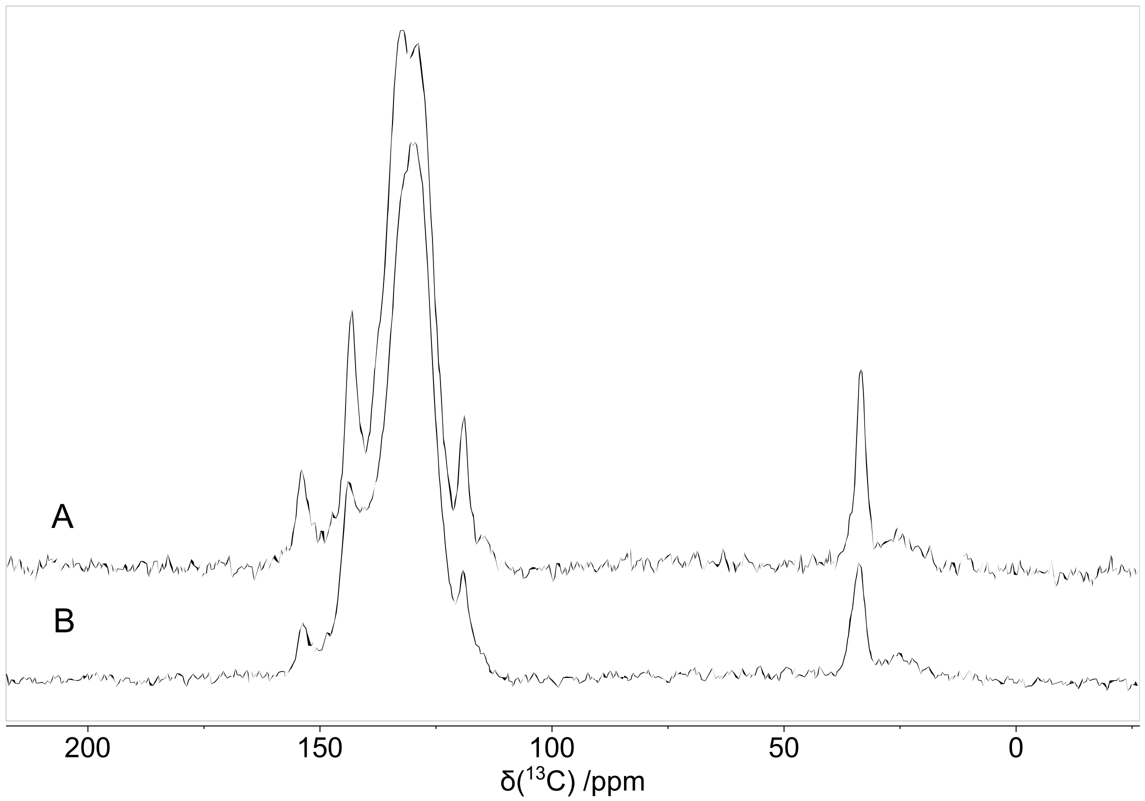

Supplementary Figure 1. $^{13}C\{^{1}H\}$ CP MAS NMR spectra of (A) GTUB5 and (B) GTUB5 after annealing at 220 °C.

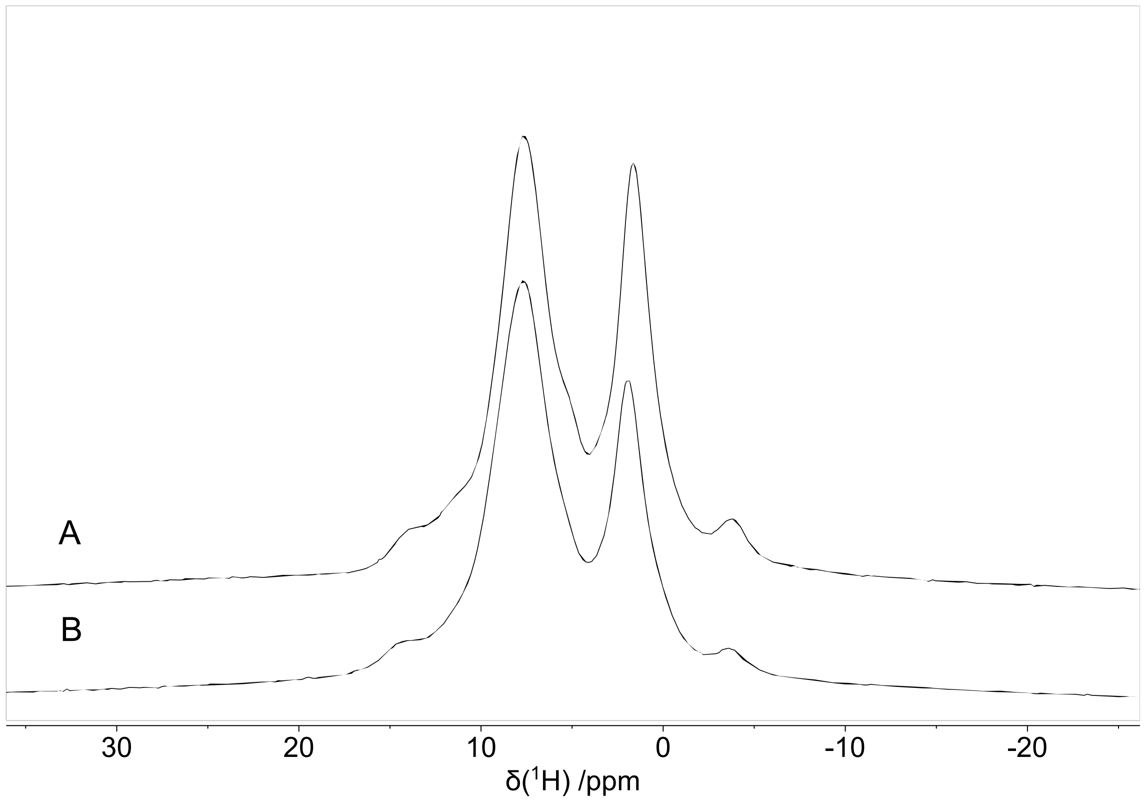

Supplementary Figure 2. ^{1}H MAS NMR spectra of (A) GTUB5 and (B) GTUB5 after annealing at 220 °C.

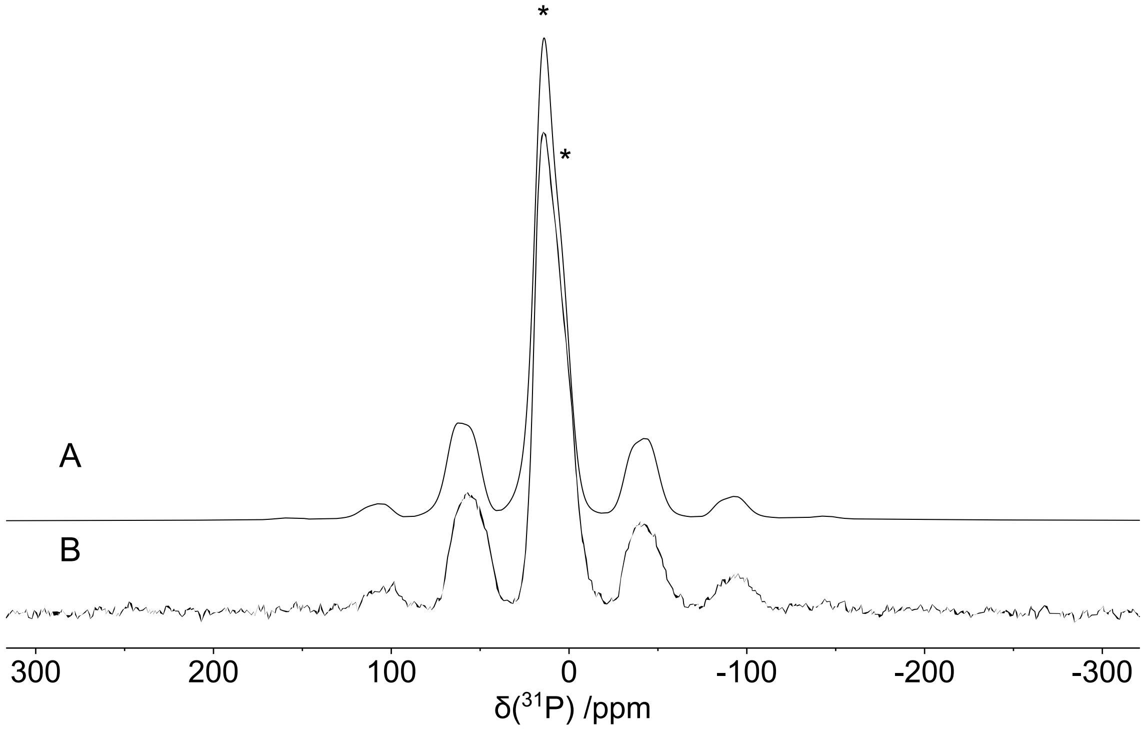

Supplementary Figure 3. Experimental (B) and simulated (A) ^{31}P MAS NMR of the GTUB5 sample after annealing at 220°C at a magnetic field of 14.1 T and a sample spinning frequency of 12 kHz.

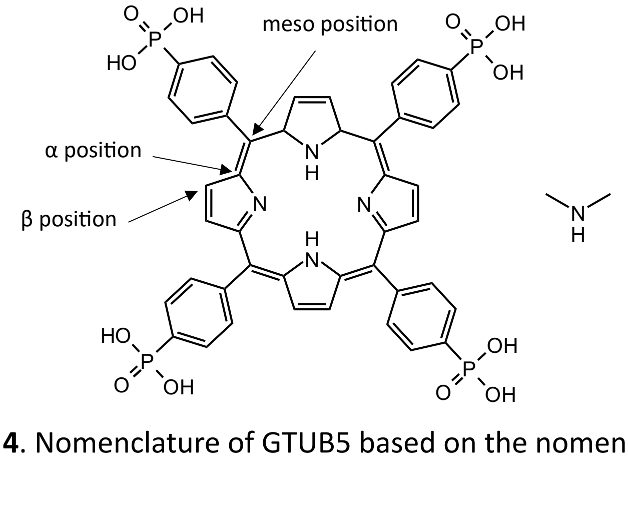

Supplementary Figure 4. Nomenclature of GTUB5 based on the nomenclature of porphyrin.

Supplementary Table 1. Isotropic chemical shift values by ^{1}H MAS NMR of GTUB5 before and after annealing at 220 °C (**Supplementary Figure 1**)

**Environments**	**δ_{iso}/ppm**
Ph	~7.7
N-CH_3	1.7
N-H	-3.8
H_β	~11
P-OH	11.0, 14.0

Supplementary Table 2. Isotropic chemical shift values of GTUB5 by ^{13}C MAS NMR before and after annealing at 220°C (**Supplementary Figure 2**) and peak assignment.

**Environment**	**δ_{iso}/ppm**
Ph	~130
N-CH_3	34
C_{meso}	119
C_α (C=N-C)	153
C_α (C-NH-C)	~130
C_β (C=N-C)	143
C_β (C-NH-C)	~130

Supplementary Table 3. The result of a non-linear least square fit of the ^{31}P MAS spinning spectrum using the SIMPSON simulation package[1] in combination with home-written fitting libraries of a sample of GTUB5 annealed at 230 °C.

	Peak A	Peak B
δ_{iso}/ppm	14.8	5.0
δ_{xx}/ppm	67.8	89.4
δ_{yy}/ppm	28.5	13.1
δ_{zz}/ppm	-52.0	-87.4
δ_{aniso}/ppm	-66.8	-92.4
η	0.59	0.83

Supplementary Table 4. The **^{31}P** chemical shift values of a series of phosphonic acids and pyrophosphate from literature.

	R -PO_3H_2 [2]	R-P(OOH)-O-P(OOH)-R [3]	-[-O-P(O)OH-]$_n$- [4,5]	
R	δ_{iso}/ppm	δ_{iso}/ppm	$\delta_{iso}(Q^1)$/ppm	$\delta_{iso}(Q^2)$/ppm
Ph	15.7	7.1	-11	-22
$PhCH_2$	24.1	17.1		
$PhCH_2CH_2$	27.7	20.7		
PhCH=CH	16.7	7.1		
$PhC(CH_2)$	14.0	5.4		

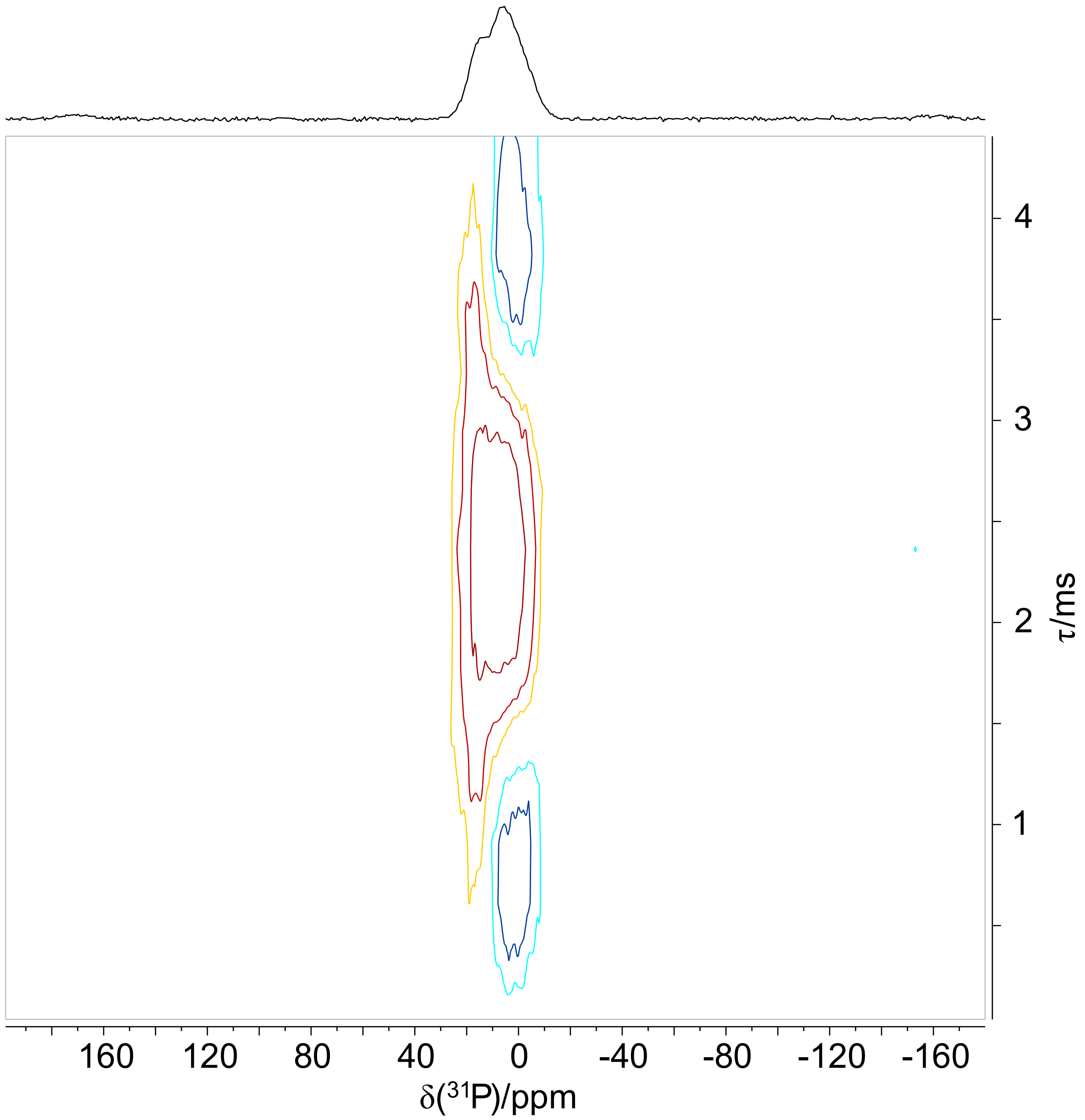

Supplementary Figure 5. Contour level plot of a ^{31}P double-quantum constant-time (DQCT) MAS NMR experiment of GTUB5 after annealing at 220 °C. The phase-adapted PostC7 pulse sequence was used at a spinning frequency of ν_r = 14286 Hz with a 7.04 T magnet and with a total double-conversion time of 4.48 ms.[6,7] The signal assigned to the monophosphonate groups does not show a zero crossing.

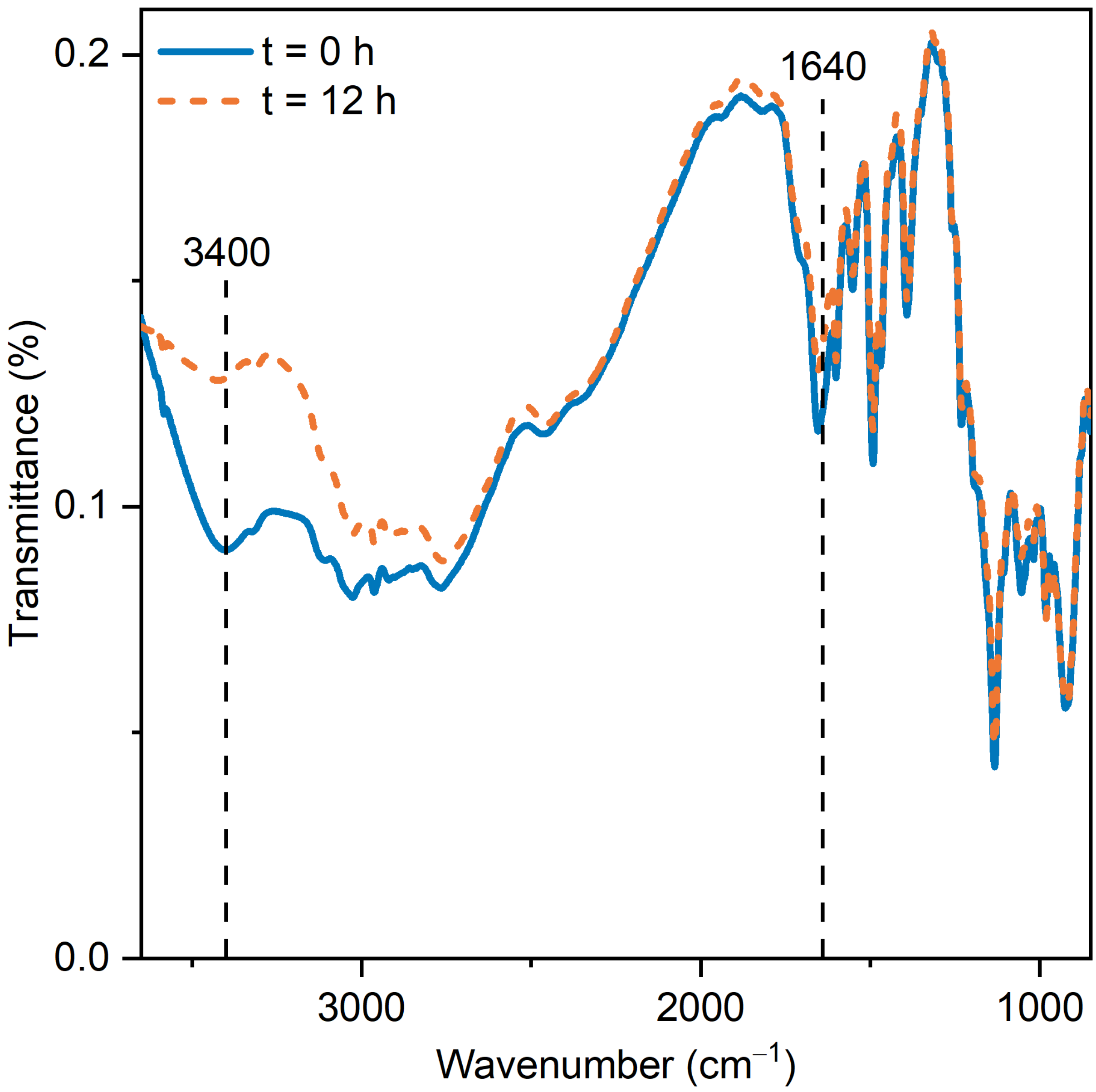

Supplementary Figure 6. Transmission spectra of GTUB-5 HOF at 50 °C (blue) and at 50 °C after 12 h in vacuum (orange).

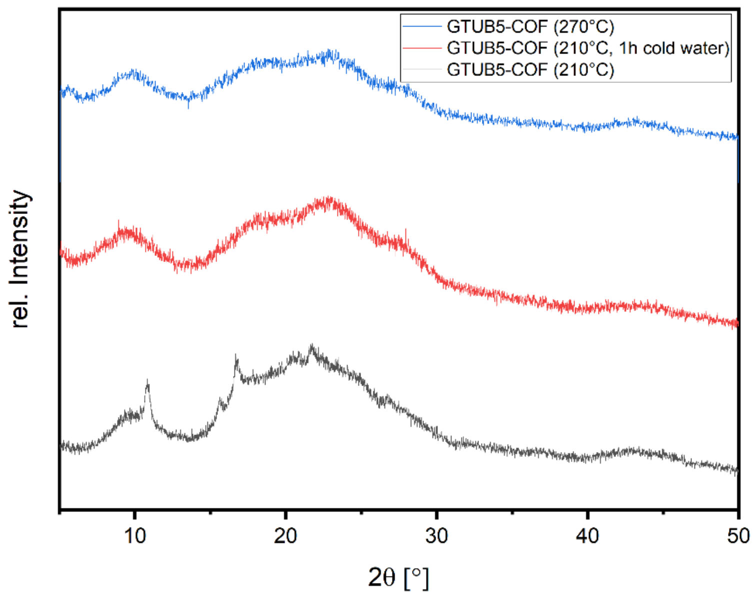

Supplementary Figure 7: Powder X-ray diffractograms measured from 5° to 50° 2θ after heating to 210 °C and 270 °C, and subsequent test in cold water for 1 h.

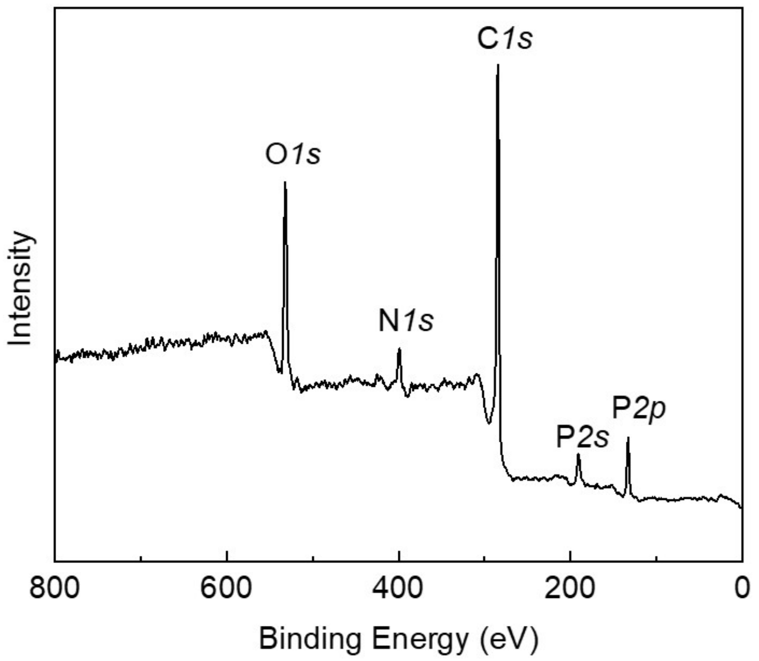

Supplementary Figure 8. X-ray photoelectron spectra of GTUB5 heated to 270. °C .

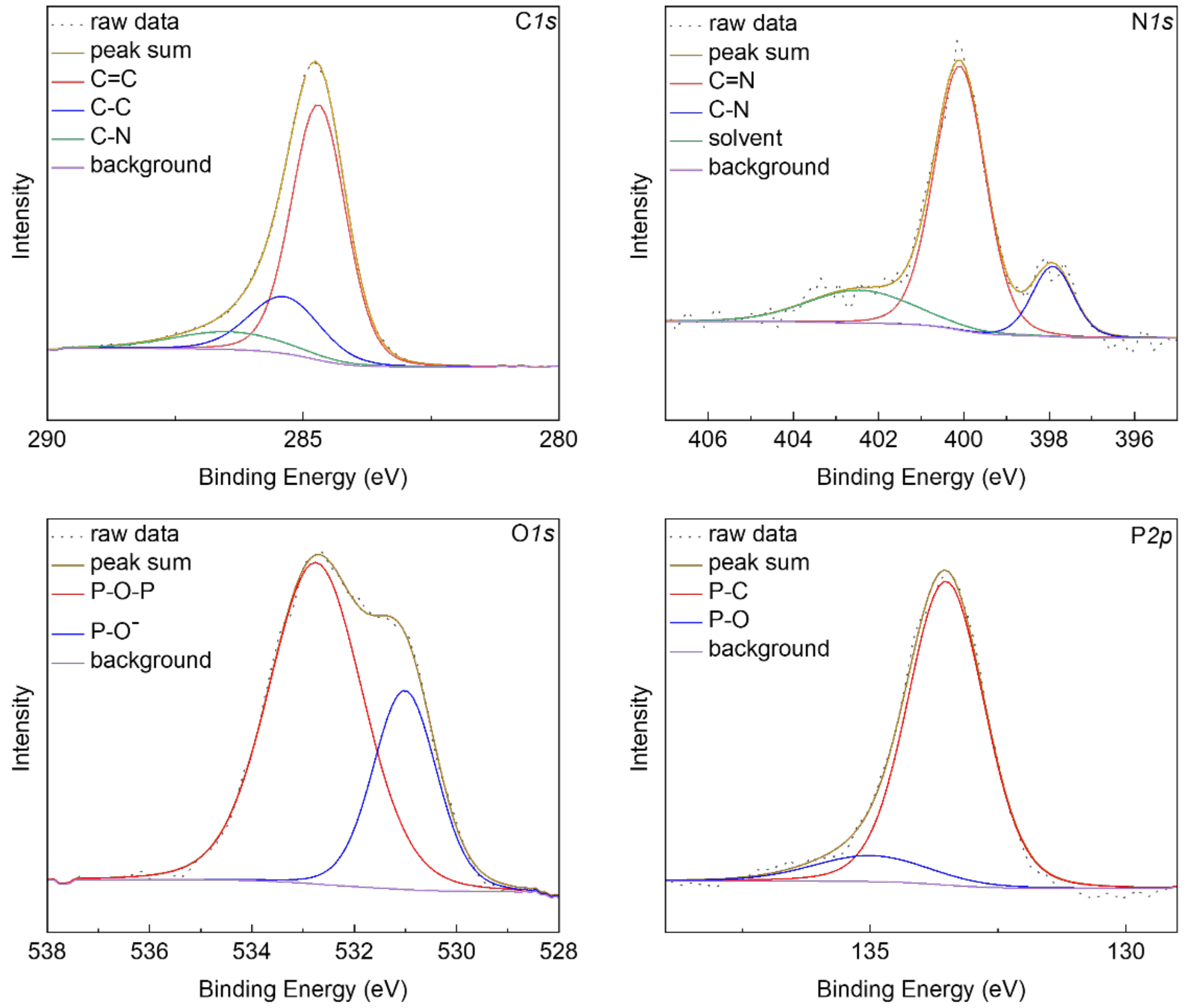

Supplementary Figure 9. High resolution X-ray photoelectron spectra of C *1s*, N *1s*, O *1s*, and P *2p* of GTUB5.

Supplementary Table 5. XPS peak types and corresponding binding energies of carbon in GTUB5.

Element	Characteristic peak	C=C (eV)	C-C (eV)	C-N (eV)
C	C *1s*	284.5	284.8	286

Supplementary Table 6. XPS peak types and corresponding binding energies of oxygen in GTUB5.

Element	Characteristic peak	P-O-P (eV)	$P\text{-}O^-$ (eV)
O	O *1s*	532.7	531

Supplementary Table 7. XPS peak types and corresponding binding energies of nitrogen in GTUB5.

Element	Characteristic peak	C=N (eV)	C-N (eV)	Solvent (eV)
N	N *1s*	400	397	402.4

Supplementary Table 8. XPS peak types and corresponding binding energies of nitrogen in GTUB5.

Element	Characteristic peak	P-C (eV)	P-O (eV)
P	P *2p*	133.5	134.9

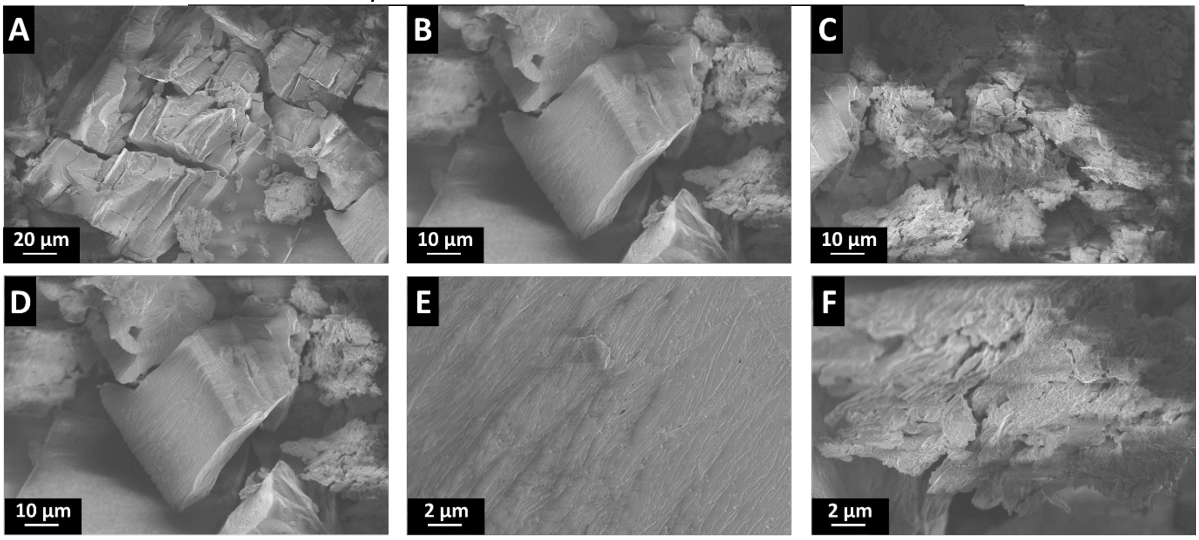

Supplementary Figure 10. Scanning electron micrographs of GTUB5-COF heated after 270 °C in different magnifications. A) 20 µm, B) 10 µm, C) 10 µm from depicting the right side of B, D) 10µm, E) 2µm view of B, F) 2µm view of C.

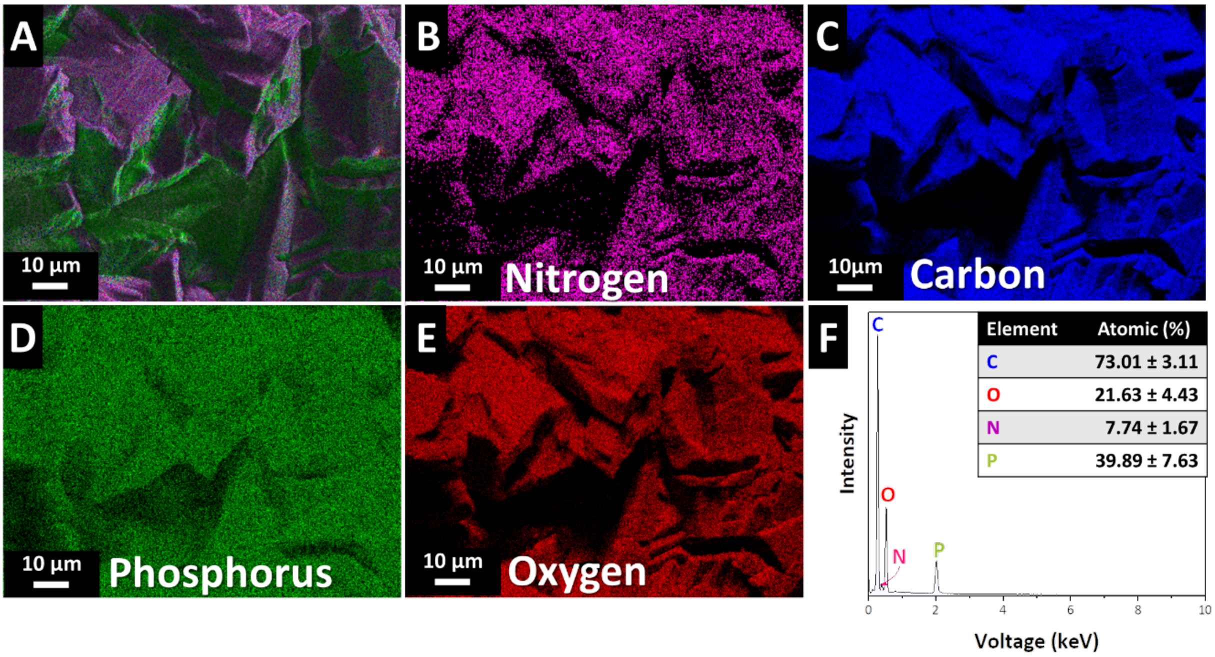

Supplementary Figure 11. Energy dispersive X-ray spectroscopy mapping of GTUB-COF heated 270 °C in different magnifications. A) Region mapped, B) Nitrogen mapping, C) Carbon mapping, D) Phosphorous mapping, E) Oxygen mapping, and F) Elemental analysis from 25 points collected. Inset: Atomic ratio of elemental analysis.

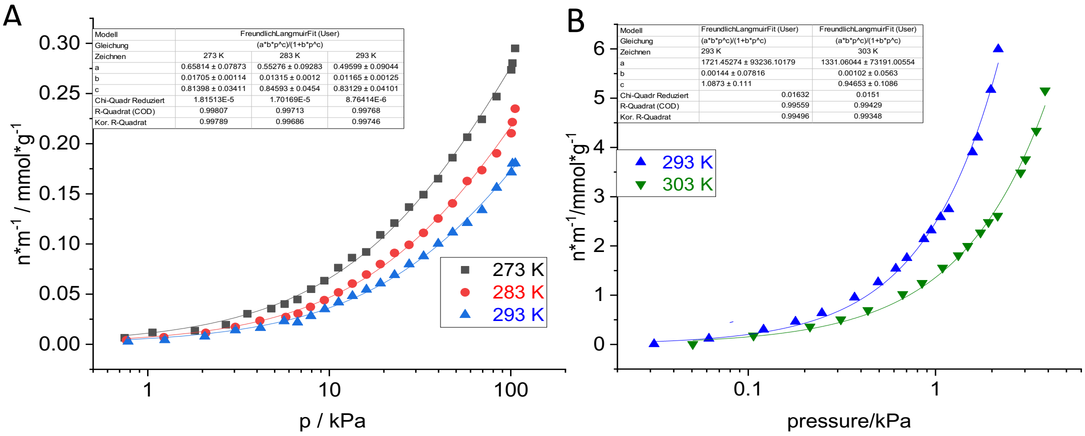

Supplementary Figure 12. A) CO_2 adsorption isotherms at different temperatures with Freundlich-Langmuir-Fit and corresponding equations. B) Water adsorption isotherms at different temperatures with Freundlich-Langmuir-Fit and corresponding equations.

Supplementary Table 9. Selected COFs from the literature with respect to their stabilities and heat of adsorption. The data are taken from references [8-10]

COF	CO_2 adsorption at 273 K and 0.95 bar (cm^3g^{-1})	Enthalpy of adsorption of CO_2 ($kJmol^{-1}$)	Stability tests
GTUB-5	6	-27	Cold and hot water; heat
TEPA(10)-COF-1	58	-37.5	Storage under humid air
POSS-TPA-COF	58	-26.3	Heat; water, acid, base, organic solvents
CO_2-COF	23	Not given	Heat; humidity
CTF-1	55	-31	Humidity

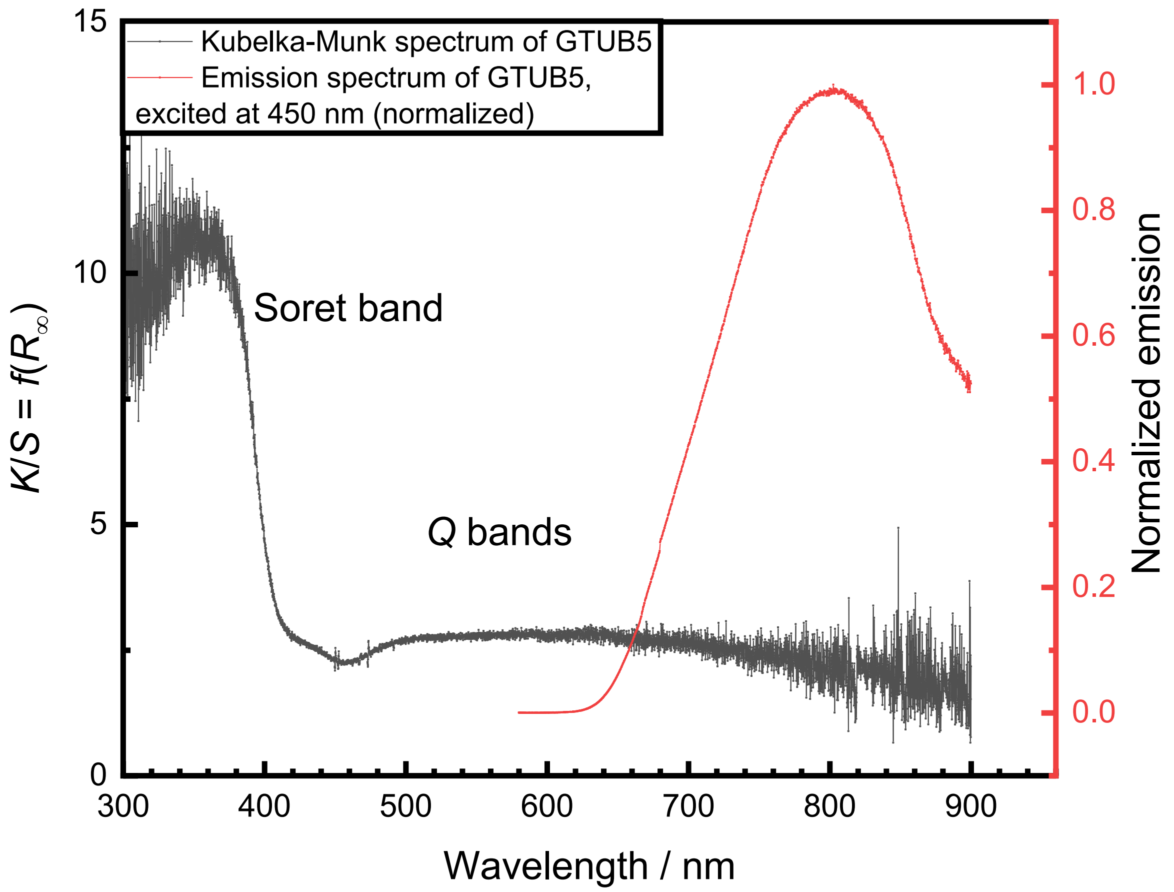

Supplementary Figure 13. Optical spectra of GTUB5 at 270 °C. Kubelka-Munk spectrum (black) as derived from diffuse reflectance spectra at room temperature and emission spectrum (excited at 450 nm at room temperature) of GTUB5 crystals heated at 270 °C.

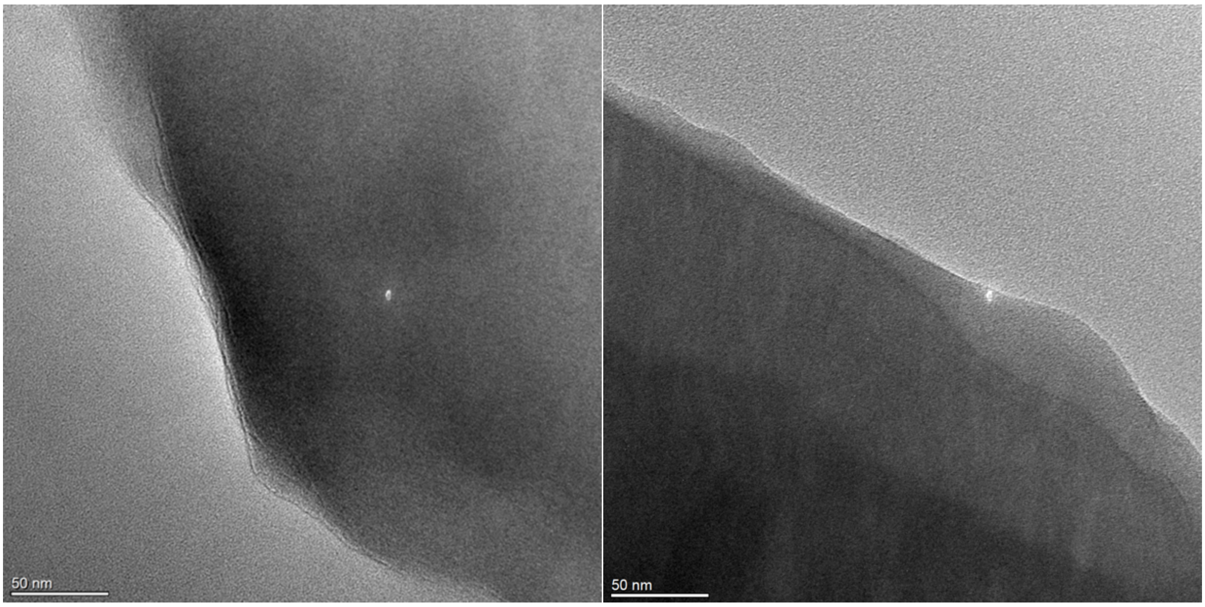

Supplementary Figure 14. Transmission electron micrographs of GTUB5 heated to 270°C.

Supplementary Information References

(1) Bak, M., Rasmussen, J. T., Nielsen, Simpson, N. C. A General Simulation Program for Solid-State NMR Spectroscopy. *Journal of Magnetic Resonance*, 147, 296-330 (2000). DOI: https://doi.org/10.1006/jmre.2000.2179.

(2) Han, X.-H., Gong, K.; Huang, X., Yang, J.-W., Feng, X., Xie, J., Wang, B. Syntheses of Covalent Organic Frameworks via a One-Pot Suzuki Coupling and Schiff's Base Reaction for C_2H_4/C_3H_6 Separation. *Angewandte Chemie International Edition*, 61, e202202912 (2022), https://doi.org/10.1002/anie.202202912.

(3) Abuzeid, H. R., El-Mahdy, A. F. M., Kuo, S.-W. Covalent organic frameworks: Design principles, synthetic strategies, and diverse applications. *Giant*, 6, 100054 (2021). DOI: https://doi.org/10.1016/j.giant.2021.100054.

(4) Jessen, H. J., Dürr-Mayer, T., Haas, T. M., Ripp, A.; Cummins, C. C. Lost in Condensation: Poly-, Cyclo-, and Ultraphosphates. *Accounts of Chemical Research*, 54, 4036-4050 (2021). DOI: 10.1021/acs.accounts.1c00370.

(5) Schülke, U., Kayser, R., Neumann, P. Zur Darstellung von Cyclophosphaten, Cyclophosphatophosphonaten, Diphosphonaten und Diphosphiten in Harnstoffschmelzen. *Zeitschrift für anorganische und allgemeine Chemie*, 576, 272-280 (1989). DOI: https://doi.org/10.1002/zaac.19895760131

(6) Weber, J., Seemann, M., Schmedt auf der Günne, J. Pulse-transient adapted C-symmetry pulse sequences. *Solid State Nuclear Magnetic Resonance*, 43-44, 42-50 (2012). DOI: https://doi.org/10.1016/j.ssnmr.2012.02.009.

(7) Schmedt auf der Günne, J. Distance measurements in spin-1/2 systems by ^{13}C and ^{31}P solid-state NMR in dense dipolar networks. *Journal of Magnetic Resonance*, 165, 18-32 (2003). DOI: https://doi.org/10.1016/S1090-7807(03)00242-8.

(8) Jia, C., Liang, R.-R., Gan, S.-X., Jiang, S.-Y., Qi, Q.-Y., Zhao, X. Boosting Hydrostability and Carbon Dioxide Capture of Boroxine-Linked Covalent Organic Frameworks by One-Pot Oligoamine Modification. *Chemistry – A European Journal*, 29, e202300186 (2023). DOI: https://doi.org/10.1002/chem.202300186

(9) Qiao, G.-Y., Wang, X.; Li, X., Li, J., Geng, K., Jin, E., Xu, J.-J., Yu, J. Unlocking Synthesis of Polyhedral Oligomeric Silsesquioxane-Based Three-Dimensional Polycubane Covalent Organic Frameworks. *Journal of the American Chemical Society*, 146, 3373-3382 (2024). DOI: 10.1021/jacs.3c12650.

(10) Zhang, S., Lombardo, L., Tsujimoto, M., Fan, Z., Berdichevsky, E. K., Wei, Y.-S., Kageyama, K., Nishiyama, Y., Horike, S. Synthesizing Interpenetrated Triazine-based Covalent Organic Frameworks from CO_2. *Angewandte Chemie International Edition*, 62, e202312095 (2023). DOI: https://doi.org/10.1002/anie.202312095.
